# Anthropomorphism in Human–Robot Co-evolution

**DOI:** 10.3389/fpsyg.2018.00468

**Published:** 2018-03-26

**Authors:** Luisa Damiano, Paul Dumouchel

**Affiliations:** ^1^Epistemology of the Sciences of the Artificial Research Group, Department of Ancient and Modern Civilizations, University of Messina, Messina, Italy; ^2^Graduate School of Core Ethics and Frontier Sciences, Ritsumeikan University, Kyoto, Japan

**Keywords:** affective coordination, anthropomorphism, social AI, social robotics, synthetic anthropology, synthetic ethics

## Abstract

Social robotics entertains a particular relationship with anthropomorphism, which it neither sees as a cognitive error, nor as a sign of immaturity. Rather it considers that this common human tendency, which is hypothesized to have evolved because it favored cooperation among early humans, can be used today to facilitate social interactions between humans and a new type of cooperative and interactive agents – social robots. This approach leads social robotics to focus research on the engineering of robots that activate anthropomorphic projections in users. The objective is to give robots “social presence” and “social behaviors” that are sufficiently credible for human users to engage in comfortable and potentially long-lasting relations with these machines. This choice of ‘applied anthropomorphism’ as a research methodology exposes the artifacts produced by social robotics to ethical condemnation: social robots are judged to be a “cheating” technology, as they generate in users the illusion of reciprocal social and affective relations. This article takes position in this debate, not only developing a series of arguments relevant to philosophy of mind, cognitive sciences, and robotic AI, but also asking what social robotics can teach us about anthropomorphism. On this basis, we propose a theoretical perspective that characterizes anthropomorphism as a basic mechanism of interaction, and rebuts the ethical reflections that *a priori* condemns “anthropomorphism-based” social robots. To address the relevant ethical issues, we promote a critical experimentally based ethical approach to social robotics, “synthetic ethics,” which aims at allowing humans to use social robots for two main goals: self-knowledge and moral growth.

## Introduction

The idea of social robots has been inseparable from that of robots since its inception. In Karel Čapek’s 1920 play “*R.U.R. (Rossum’s Universal Robots)*,” from which science and engineering inherited the term, the human-like artifacts called “robots” are artificial social agents that function as secretary, postman or factory workers (Čapek, 1920/2004). Compared to these fictional ancestors, today’s social robots are quite different. For one, they are not bio-chemical, but mechanical artificial agents. Moreover, their social abilities do not arise spontaneously, as an apparent secondary effect of success at biochemically re-creating life. Generating the social skills of mechanical robots requires from actual “social robotics” (SR) highly specialized research in a variety of fields, original design and a complex process of implementation (Fong et al., 2003). For the goal of SR is not to produce mere tools. Specialists in SR intend to build artificial agents capable of social performances that, in the perspective of their human users, can make them rise above the status of instruments to that of interlocutors (Kaplan, 2005). In a sense, this goal remains true to Čapek’s fictional ideal of creating “artificial workers” engaged in a broad range of services – information, education, coaching, therapeutic mediation, assistance, entertainment, and companionship, among others. However, SR acknowledges that, to perform in these fields, robots need to exhibit many social behaviors and, in particular, to evince a believable “social presence,” defined as a robot’s capability to give the user the “sense of being with another” (Biocca et al., 2003), or the “feeling of being in the company of someone” (Heerink et al., 2008). It is here that SR most interestingly departs from the imaginary project that was at the heart of R.U.R.’s fictional robotics. Chapekian robots were almost perfect bio-chemical copies of humans, but Rossum had made slaves, ‘animated instruments,’ whose sociality he negated in an unsuccessful attempt to reduce these subjects to mere objects. SR aspires to do exactly the opposite: to allow mechanical objects to play the role of subjects, devising artificial agents that will not only be “tools,” but also act as “social partners” (Dumouchel and Damiano, 2017). It is in the context of this project that we propose to consider the complex relationships between SR and anthropomorphism.

## Social Robotics as Applied Anthropomorphism

### Reevaluating Anthropomorphism

Anthropomorphism is generally understood as the human tendency to attribute human traits to non-human entities (Epley et al., 2007; Złotowski et al., 2015), or to treat “non-human behavior as motivated by human feelings and mental states” (Airenti, 2015). As such, traditionally it has been viewed as a bias, a category mistake, an obstacle to the advancement of knowledge, and as a psychological disposition typical of those who are immature and unenlightened, i.e., young children and “primitive people” (e.g., Caporael, 1986; Fisher, 1996; Mitchell, 2005). In contrast to this traditional negative evaluation, SR grants anthropomorphism a positive, and plurally articulated, role. The fact is that the tendency to anthropomorphize is quite frequently manifest among humans, and thus the goal of building social robots suggests that it may be used as a tool to facilitate social exchange between robots and humans. The underlying idea is to actively involve users in the social performances and presence of the robots, by designing robotic agents that stimulate users to attribute human feelings and mental states to robots, which should enhance familiarity and promote social interactions. However, if anthropomorphism is an infantile and primitive character trait, the question arises: is it legitimate for SR to exploit what must essentially be viewed as a human failing?

It should be noted that, though it persists in more or less ambiguous forms (e.g., Caporael, 1986; Kennedy, 1992; Mitchell, 2005; Wynne, 2007), the negative evaluation of anthropomorphism has received challenges from many disciplines. For example, evolutionary anthropology and the cognitive science of religion developed a more positive conception of anthropomorphism as a cognitive device that augmented human fitness. It is argued that the tendency to see human faces or bodies in ambiguous shapes provided important fitness advantages to early humans, helping them to distinguish between friends and enemies, to rapidly recognize predators, and to establish alliances with other tribes (Guthrie, 1995; Bering, 2005). Anthropomorphism would then be an evolutionary adaptation that, according to many authors, is inseparable from religion and is often associated with the existence of a Hyperactive Agency Detection Device (HADD) (Barrett, 1998; Westh, 2009). This re-evaluation of anthropomorphism is reinforced by recent findings in cognitive sciences, which question its classic (for example, Piagetian) psychological understanding that confines anthropomorphism to the early childhood, and essentially views it as a cognitive mistake (Airenti, 2015). This new conception argues that anthropomorphism constitutes a fundamental and permanent dimension of the human mind, rather than an early stage of its cognitive development, that is grounded in neural mechanisms also found in other older species, and which is modulated by individual traits (Duffy, 2003; Złotowski et al., 2015; Levillain and Zibetti, 2017).

### Modulating Anthropomorphism

In order to successfully utilize anthropomorphism, SR has been exploring its underlying mechanisms, and how interactive robots can trigger and regulate them. Therefore, a significant part of its research enquires into the conditions of activation of anthropomorphic projections. The focus is on two key factors, human-like (anthropomorphizing) appearance and autonomous movement or behavior (Levillain and Zibetti, 2017). The basic hypothesis is that strong realism *in either* of these two factors allows a robot to reach the “social threshold” where humans experience its presence as that of another social agent and are disposed to socially interact with the machine. This implies that a highly anthropomorphic robot can produce that social effect even when behavioral realism is low, and, vice versa, that behavioral realism will lead to anthropomorphic projection even in the absence of a human-like appearance. Things, however, are not quite that simple, in particular, the relation between the two factors appears to be asymmetrical. When the threshold is reached in result of human-like appearance only, and the movements or behavior of the robots prove inadequate – that is, inconsistent with the anthropomorphic projection – a sudden “non-linear” effect in terms of familiarity and social interaction will take place. In robotics, the best-known example of this is “the Uncanny Valley” effect hypothesized by Mori (1970), in which an increase of human-likeness raises a robot’s likeability until the resemblance becomes nearly perfect. At that point, conjectures Mori, takes place a strong negative emotional reaction and a rejection of social interaction; the robot strikes its human partner as a strange, uncanny object. This sudden change, as we argued elsewhere, is the result of a mismatch between resemblance and movement – a dissonance stemming from unrealistic movements and behavior in a highly human-like robot (Dumouchel and Damiano, 2017). Furthermore, as experimental findings show, the discrepancy between resemblance and movement functions in the opposite direction when it is, so to speak, inverted. When there is little or no human resemblance but high behavioral realism, the effect on likeability and social presence tends to be positive. When any object begins to manifest, for example, autonomous coordination with a human’s movement, the person is inclined to socially interact with the object even in absence of human-like appearance. These results, consistent with empirical evidence from psychology (Urquiza-Hass and Kortschal, 2015), suggest that realistic behavior dominates human-likeness in activating anthropomorphic projections.

### ‘Good’ Anthropomorphism: Ascribing vs. Inferring

Research in SR tends to emphasize the plural articulation of anthropomorphism (Duffy, 2003). Interestingly, it stresses the difference between the form of anthropomorphism occasioned when one interacts with social robots, and anthropomorphic projections evoked by other types of objects, such as traditional dolls, cars or computers (Levillain and Zibetti, 2017). This difference is generally described in terms of the cognitive activity involved. In the second case, the subject *ascribes* human traits to non-human entities, while, in the case of social robots, *infers* these traits from the behavior of the non-human entity. Note that this distinction partially overlaps the difference between the two factors analyzed above: on the one hand, the static human-like (anthropomorphizing) appearance of the robot seems to correspond to the simple ascription of human traits, while, on the other, the dynamic realism of autonomous movement appears as the basis of the inference from behavior.

This distinction between different forms of anthropomorphism is also important to understand the ambiguous relationship that SR entertains with anthropomorphism. Inasmuch as it is inseparable from a comparative evaluation of the two forms, this distinction reveals a partial re-alignment with the negative attitude that remains dominant in science (Złotowski et al., 2015). Projected anthropomorphism is viewed as based on a fallacy and receives a negative evaluation, while anthropomorphism that is inferred from, or triggered by, the autonomous behavior of robots is positively evaluated. It is argued that it is based on empirical evidence which provides a potentially plausible explanation of the phenomenon. Notwithstanding this mildly self-serving argument – SR rests on anthropomorphism, but only on the ‘good one’ – the distinction corresponds to a valorization of the research and technical efforts dedicated to creating social robots, and to an attempt to determine the difference between common artifacts and the anthropomorphizing machines that are social robots.

### Applied Anthropomorphism: Social AI

The project of endowing robots with social traits, or making them able of social “performances,” does not require robots to understand the performed task, nor to have the social “competences” and “properties” that underlie this understanding (Pfeifer and Scheier, 1999). If this project takes its origins in classic AI, the development of a particular field of robotics dedicated to creating social robots was strongly influenced by the “Embodiment turn” in the cognitive sciences (Damiano et al., 2015; Dumouchel and Damiano, 2017). This supposedly ‘paradigmatic shift,’ which emphasized the role and importance of the body and of the environment in the cognitive competence of agents, also led to giving greater attention to the social environment as a fundamental factor in cognitive competences and development. In consequence, emerged within SR a new approach to artificial intelligence that can be defined as ‘social AI’ – and not simply “artificial social intelligence.” Its goal is not merely to artificially reproduce the ‘social intelligence’ of human agents. Indeed, its central claim is that human intelligence is essentially social. The roots of this hypothesis go back to a well-established trend in the cognitive sciences (Humphrey, 1976) and primatology (de Waal, 1982; Byrne and Whiten, 1988) arguing that human intelligence emerged from the need of solving ‘social problems.’ However, over the years it abandoned its early focus on deception and manipulation, which characterized it when it was named “Machiavellian Intelligence.” It granted growing importance to the role of cooperation characteristic of human intelligence and social interactions, as opposed to other primates (Chapais, 2008; Tomasello, 2008; Hrdy, 2009). The procedure adopted in social AI is to use human social competences, and the interactive and cooperative dimension of human intelligence, as models to develop similar performances and abilities in robotic agents. These attempts at tailoring on ours the social and cognitive performances of robotic agents are equivalent to attributing human traits to robots by implementing them. This ‘applied anthropomorphism’ inverts the metaphor that guided classic AI for more than 50 years. Rather than seeing in the computer the model of the human mind, SR uses human social and cognitive competences as a model for the social and cognitive performances of artificial social agents. Finally, the applied anthropomorphism of SR typically constitutes a synthetic approach (Pfeifer and Scheier, 1999) to the subject.

### Anthropomorphism as a Method: ‘Synthetic Anthropology’

Exploiting different combinations of these various forms of anthropomorphism, SR produced a wide range of artificial social agents. They can be seen as belonging to a ‘triangular spectrum,’ whose vertexes can be exemplified with three kinds of robots: (i) robots like Paro^1^, whose realistic animal-like appearance encourages anthropomorphic projections, in spite of its limited social AI; (ii) robots like Jibo^2^, whose appearance is not conducive to anthropomorphism, but which nonetheless gives rise to such projections because of its sophisticated social performances; and (iii) robots like Affetto^3^, whose anthropomorphic appearance is matched by high level social AI. It is important to note that all social robots, independently of where they are located on this spectrum, tend to reach the threshold at which, in the eye of the user, objects become subjects. This is indicated, or at least strongly suggested, by research on human users’ representations of social robots (Kahn et al., 2002; Severson and Carlson, 2010; Turkle, 2011; Gaudiello et al., 2015). Empirical results show that social robots tend to blur the traditional ontological categories that humans use to describe the world. More precisely, these results show that not only children, but also teenagers, adults and the elderly perceive social robots as ambiguous objects, which transgress the boundaries of traditional ontological categories and dichotomies. They are viewed neither as “sentient” nor as “not sentient,” neither as “intelligent” nor as “not intelligent,” neither as “alive” nor as “not alive” (Kahn et al., 2002). According to researchers, interactive computational technologies bring people to revise the ontological categories they use to classify objects that, like social robots, are located somewhere in between the terms of the old dichotomies – objects that are “sort of alive” or “alive enough” (Turkle, 2011). Human users attribute them a status that is somewhere in between, one that does not clearly fall on either side of these dichotomies.

The ambiguous status of social robots became the origin for a new scientific endeavor, whose relevance grows as the comparison between humans and social robots yields ever more ambivalent results. As the frontier between humans and robots is progressively blurred, the question of what constitutes human identity, or particularity, is raised anew. On this basis, anthropomorphizing robots make possible a novel science of human beings (Parisi, 2014), in which they (robots) function as both ‘objects’ and as ‘instruments’ of an inquiry about “what is human?” (Kahn et al., 2007). The central idea is that of an innovative comparative ethology and psychology. Instead of trying to understand the human species through its similarities and differences with other animal species (Tomasello, 2008), this new comparative science uses as terms of comparison the changing abilities of robots. Hiroshi Ishiguro’s “android science” (Ishiguro, 2006; MacDorman and Ishiguro, 2006; MacDorman et al., 2009) occupies a leading place in this line of research. The original inspiration, which stems from classic AI, is interpreted by the embodied approach of SR and realized through the anthropomorphic robots it builds. This offers the possibility of comparatively studying human minds as one among other “embodied minds.” Applied anthropomorphism, as practiced by SR, thus acquires the position of the central research method of a new science of human beings. This is a kind of ‘synthetic anthropology’ that promises to expand our knowledge of ourselves through systematic comparison with our increasingly sophisticated doubles.

Despite the unquestionable scientific interest of this new research direction, related technological applications in SR raises questions. Current literature emphasizes how highly human-like robots can be perceived as menacing by users, especially when they appear able to perform better than humans (Yogeeswaran et al., 2016) and display autonomy (Złotowski et al., 2017). According to the “threat to distinctiveness hypothesis” advanced by Ferrari et al. (2016), the increasing blur of boundaries between robots and humans destabilizes the perception of “human uniqueness,” and tends to generate growing concern on the negative impacts of this technology (Ferrari et al., 2016).

### Ethics: The Anthropomorphic Imposture of Social Robots

The anthropomorphism of social robots is considered to entail a variety of dangers, which span, for (vulnerable) users, from cognitive and psychological damages to manipulability and reduced quality of life^4^ (Lin et al., 2012, e.g., chapters 4, 12, and 15). Among these criticisms, there is an ethical concern that denounces the use of anthropomorphism to create social bonds between humans and robots, and judges it unacceptable. This denouncement, which rejects and condemns the applied anthropomorphism central to SR’s project, acquires relevance in that it orients current attempts to ethically regulate robotics^5^.

Sherry Turkle, among those who extensively investigated human–robot interaction through ethnographic research, is one of the most eminent voices of the ethical concerns raised by anthropomorphic robots. She grounds her argument on two important dimensions of social robots. First, to the extent that they are “relational artifacts,” the anthropomorphizing design of social robots presents them as “artifacts that have inner states of mind” and interacting with them is assumed to involve “understanding these states of mind” (Turkle, 2005, p. 62). Second, ethnographic studies focusing on children and the elderly indicate that social robots are also “evocative artifacts,” which foster the emergence of affective bonds that users tend to describe as reciprocal love and care. Anthropomorphizing robots, argues Turkle, presses our “Darwinian buttons”; they activate responses typically related to strong affective relations, such as the nurturing instinct in children, or memories of old loves in the elderly. On this basis, they mobilize high emotional charges and create an “illusion of relationship” (Turkle, 2007, 2011 p. 514). The main idea of Turkle’s ethical criticism of the use of anthropomorphism in SR is that social robots constitute a form of “cheating” technology. Their anthropomorphizing characteristics tend to falsely convince their users – especially the most vulnerable ones – that they can provide *real* social relations, with *genuine* and *reciprocal* affect and emotions, while they simply cannot. Thus, Turkle sees in social robots a further step in the development of our “culture of simulation,” which threatens to turn people away from “real” social relationships – that is, from relationships with other humans – and reduce their social life to an illusion – to the feeling of being together with someone, when in fact one is alone. She concludes her radical criticism of all anthropomorphizing computational technologies by claiming that they “should not be allowed into the realm of human relationships” (Turkle, 2010, p. 4).

One interesting, and significant, aspect of this way of conceptualizing the ethical issue – which is in fact quite common – is that it relies on oppositions, for example ‘authentic/simulated’ or ‘true/false,’ which many years ago were used to question the validity of classic AI. The question that was then asked was: “Do computers really think, or do they just simulate thinking?” The efforts to answer, and to confront these dichotomies, ultimately led to the distinction between “weak” and “strong” AI. The AI that simulates and fakes it, and the AI that promises to deliver the ‘real thing.’ In relation to SR and social AI, the questions are: “Do anthropomorphizing robots expose their users to authentic or simulated social behavior? Is love expressed by a robot ‘real love’, or is it ‘simulated love’?” Turkle’s answer is that “simulated thinking may be thinking, but simulated feeling is never feeling, simulated love is never love” (Turkle, 2010, p. 4). We believe that SR’s applied anthropomorphism both allows and requires us to address these questions, as well as the ethical concerns raised by social robots, in a different way.

## Anthropomorphism and Social Coordination

### Anthropomorphic Projections as Action

Anthropomorphism, as applied by SR, challenges the traditional understanding of the phenomenon in a variety of ways. Rather than seeing it as a cognitive mistake, SR views anthropomorphism as a fundamental tool in successful human–robot relations. Rather than condemning anthropomorphism as an unjustified attribution of mental states to inanimate objects, SR exploits it to create artificial agents that challenge the subject/object divide. However, as we have just seen, anthropomorphism, in its traditional form, comes back to haunt SR as the ethical criticism of the design and use of social robots. Implicit in that criticism is the conviction that anthropomorphic projections correspond to false beliefs. The mistake involved can be benign when the commitment to the false belief underlying the projection is weak. For example, when we say: “the weather doesn’t want me to go shopping today.” Come to think about it, we do not *really* believe that the storm *wants* anything. However, Turkle and others argue that this mistake can have important consequences when the false belief becomes entrenched or gains strong motivational force. For example, when children believe their robotic caregiver sincerely cares for them, the danger, according to Sharkey and Sharkey, is that robots tend to exploit, and even amplify, “children’s natural anthropomorphism” (Sharkey and Sharkey, 2010, p. 164).

We are not sure if robots amplify “natural anthropomorphism” or not. We certainly agree that SR exploits it, as our arguments in the previous sections show. However, we do not think that “natural anthropomorphism” is proper to children, nor that it is or rests on a cognitive mistake. Recent studies in psychology (Epley et al., 2007, 2008; Timpano and Shaw, 2013) and in neuroscience (Scheele et al., 2015) recognize that anthropomorphism is closely related to human sociality. They retain nonetheless the traditional conception of the phenomena, and consider that anthropomorphism is primarily a question of (false) beliefs. They then inquire into the social conditions – for example, lack of, or poor, social relationships – that encourage people to attribute mental states to non-human animals and objects (Paul et al., 2014). In this context anthropomorphism is viewed as a form of compensation, a way of dealing with solitude, or a reaction to the loss of a loved one – a sign that something is amiss. SR, on the opposite, considers it as a central aspect of sociality, and tries to harness its pragmatic and relational dimension.

If you ask a friend to borrow his jigsaw and, as he hands it to you, he adds “Be careful, it is a bit temperamental!”, how should you interpret this remark? It is unlikely that you will conclude that he sincerely believes that his jigsaw has moods, mental states and other psychological dispositions. If you do, you will have misunderstood the nature of the interaction. The point is not that this use of language is metaphorical. Rather it is that, by attributing to the other this outlandish belief, you fail to recognize what he has just done: to warn you and recommend care. Isn’t “Be careful!” enough? By adding “It is a bit temperamental!”, he directs your attention to the fact that his warning concerns the use of the jigsaw, that he is not so much worried that you will damage his machine as your own work while using it, and he recommends you to treat it gently. “Gently.” Another metaphor? The anthropomorphic use of language is not metaphorical here, because there is no corresponding literal way of saying it that would accomplish what his warning and recommendation do.

Even if it were possible to describe in detail the types of circumstances in which the jigsaw reacts strangely, the forms of its unexpected reactions, and the necessary precautions, such a list is not equivalent to a warning and recommendation. It is not an action, not a performance, but a description. While the list leaves you free to follow its indications or not, the more detailed it is, and the more it constrains your behavior, instructing you what to do. Though the your friend’s warning may exert a certain social pressure upon you, because it simply directs your attention to the “temperamental” character of the jigsaw, it leaves it up to you to find out how and when to be careful. Thus, the anthropomorphic language reaffirms what is implicit in lending you the jigsaw, that its owner trusts you. It treats you like an agent in an interaction, unlike a set of instructions that can govern a machine.

### Interacting With Agents

Anthropomorphic statements should not primarily be understood as descriptive statements, but as pragmatic statements in the context of interaction. As such the projection does not need to rest on the attribution of mental states to the anthropomorphized entity, nor imply any false belief. When someone says about her car, or computer, “It does it on purpose!”, she does not believe that her car is an intentional agent or that her computer hates her, and thus breaks down when she needs it most. What she attributes, or rather recognizes, is the changed role of these objects within the interaction. Breaking down ‘agentifies’, so to speak, the object. It transforms the object from a dependable mechanism, which regularly fulfills its function, into an agent or a subject – that is: into something whose behavior is to be explained in relation to itself. The best, and most familiar, models we have of such entities, and of interacting with them, are other humans, and social interactions. Spontaneous anthropomorphic projections take place when we discover that we are now dealing with an entity that needs to be explained in relation to itself, rather than simply in relation to our own goals and purposes. More precisely, it corresponds to the recognition that we are interacting with an entity whose behavior is, to some extent, determined by itself – an agent.

Anthropomorphic projections do not rest on the prior belief that an object or animal has human like mental states. It rests on the recognition that one is dealing with an entity that acts – even if it only ‘acted up’, so to speak – and that the relation has changed, from, say, a relation of use to a form of interaction. That is: to a relation that requires the coordination of the actions of two ‘agents’ for any one of them to be able to achieve his, her or its goal. Anthropomorphism is the recognition of ‘inter-subjectivity’ in action. Our claim then is that a large class of anthropomorphic statements are expression of the mechanism underlying what Trevarthen and Delafield-Butt (2017) describe as “primary” and “secondary inter-subjectivity”: the ability to coordinate one’s action to those of another. This ability is already present in very young infants, and does not, in any way, require the attribution of beliefs. It rests on basic neuronal mechanisms, and constitutes a fundamental building block of who we are as social and cognitive agents. According to Trevarthen, primary and secondary inter-subjectivity do not disappear as the child matures, but are integrated as necessary elements in “tertiary consciousness of inter-subjectivity.”

Anthropomorphic projections do not require, nor necessarily imply, the belief that a non-human animal or object has mental states similar to ours. Nonetheless, in many cases, they will lead to the formation of such beliefs, which may or may not be true. Historically, the term ‘anthropomorphism’ has been reserved to refer to when the attribution fails, and the belief is false. Yet, there has been, and there still is, uncertainty as to when that is the case. For example, whether or not, and to what extent, it is legitimate to attribute beliefs, desires, or emotions, like fear or loneliness, to a dog, a cat, a horse, a monkey or a lobster, are issues on which there is no universal agreement. Not so long-ago behaviorists thought that attributing mental states to human beings was unscientific, and some philosophers even argued that we should discard the mentalist language of folk psychology and replace it by one derived from neuroscience (Churchland, 1996). Yet, in action, if not in their writings, all adopted the intentional stance when interacting with others. Anthropomorphism is primarily a tool for interacting, not a description of the world.

### Affective Coordination

Turkle’s claim – “simulated thinking may be thinking, but simulated feeling is never feeling, simulated love is never love” – rests on an understanding of mind and emotion that is closely linked with the conception of anthropomorphism as false belief. This view was originally crafted by Descartes (1641/1998) and Descartes (1649/1989), and its dualist conceptual structure, in spite of repeated denials, was inherited by contemporary philosophy of mind, mainstream cognitive science and AI (Damiano et al., 2015; Dumouchel and Damiano, 2017). According to Descartes, mind and body are two radically different substances. Thought, the ‘action’ of the mind, consists in reasoning performed by an immaterial soul. In cognitive science, this soul becomes an abstract mathematical entity, and thought the computations it executes. Just as the soul transcends matter, the computational mind is indifferent to ‘that’ in which it is implemented, given the required functional equivalence is maintained. Given, to put it otherwise, that the system is implemented *as such*, or as the system that it is, the matter in which it is implemented does not matter. Thus, that artificial agents may think – “simulated thought may be thought,” as Turkle concedes – is perfectly consistent with this conceptualization of mind.

According to Descartes (1649/1989), feeling and emotions are produced by the body. They are events that take place internally, in the intra-individual ‘space,’ where the epistemic subject ‘resides.’ Thus the mind perceives – or rather experiences – them directly. In consequence, the emotion produced by the body, and experienced internally, can never be false – it is always genuine. There is, however, a second aspect of feelings and emotions: their external expression. Relative to the emotion itself, produced and experienced internally, its expression is secondary and contingent, for the subject can suppress the expression, or even fake (simulate) having an emotion he or she does not have. The expression is external: it is a public event, in inter-individual space, and others can perceive it. Here, in this social space, emotions can be either true or false, simulated or genuine, depending on the relation between the expression and the subject’s internal state.

This way, the first dichotomy, the body/mind divide, leads to a series of other dichotomies, which reproduce the original valuation that exalts the mind above the body, and computation beyond mere matter. Production/expression, internal/external, private/social, necessary/contingent, but also genuine/simulated, and true/false: whatever is on the left-hand side of the slash is deemed superior to what is on the right side. We may think that we have abandoned Descartes’s dualism. However, it is clear, from Turkle’s claim, that we did not abandon the dichotomous way of thinking we inherited from him.

Within this conceptual scheme, the emotions expressed by robots can only be false, simulated, inauthentic, because robots lack the internal emotion that is the warrant of the truth and authenticity of affective expression. Attributing feelings to social robots constitutes a form of anthropomorphism. It rests on the false beliefs that these machines have internal states that correspond to the emotion they express – an illusion that they tend to encourage.

In SR, based on the “affective loop approach” (Damiano et al., 2015), an “emotional” robot is defined by its capacity to engage users in a dynamic interaction that includes affective expressions and appropriate responses triggering further reactions on the part of both the human and its artificial partner. The goal is to make “the user [affectively] respond and step-by-step feel more and more involved with the system” (Höök, 2009), in a way that enhances the robot’s social presence and favor human–robot social interaction (Paiva et al., 2014). This goal can be achieved with either of two kinds of robots. The first kind simply expresses emotions by realistic appearance and motion. The second kind combines these expressive skills with social AI to manifest “intelligent expression,” that is, emotional expression coordinated with that of their users.

An interesting aspect of these successful implementations of the affective loop is that they violate two fundamental assumptions of the ‘Cartesian’ approach (Damiano et al., 2015). First, they do not treat the robot as an ‘individual,’ that is, an independent affective agent whose emotion is essentially internal and private. The target of the affective loop is not to produce emotions within the robotic body, but to create a recursive human–robot emotional dynamic that generates robotic emotional expressions in – more or less ‘socially intelligent’ – artificial agents. The goal is to coordinate the affective expression between human and robotic agents. The second difference is that, within this affective exchange, the robot’s expressions do not communicate pre-existing emotions. They function directly as a means of generating human emotions. They trigger immediate emotional reactions that do not need, or rest on, the complex process of interpretation which philosophy, psychology, and classic cognitive sciences postulate as necessary for a person to access others’ emotions. This affective coordination bypasses both theory of mind and folk psychology. Applied anthropomorphism does not require any false beliefs.

The robots developed by the affective loop approach illustrate a different conception of emotion, which can be traced back to Hobbes (1650/1994). Since then, it remained present, though somewhat marginal, in philosophy. Recently it has been ‘re-evaluated’ by embodied cognitive science, and received support from the discovery of mirror neurons and related mechanisms (Rizzolatti et al., 2001). This view proposes to consider affect as an evolved mechanism of coordination between agents (Dumouchel, 1999). The fundamental hypothesis is that affective expression is part of a continuous process of inter-subjective coordination, in which agents reciprocally determine each other’s emotions and dispositions to action. Within this dynamic, expression and determination of emotion are inextricably entangled, and cannot be separated. Affective expression is a direct mean of influence among agents in interaction, which contributes to the mutual specification of their dispositions to act. Far from engaging in a rational calculus (or simulation) aimed at discovering the emotions of others, agents participating in inter-subjective interactions directly co-determine each other’s emotional state. Recent results suggest that this process of emotional co-definition may be supported by “mirroring mechanisms,” which do not only couple perception and action, but also perception and the expression of emotion. Indeed, mirror neurons fire not only when a subject expresses an emotion, but also when he or she observes another person expressing it.

Within this different conception of emotions, the oppositions that are commonly used to understand and evaluate emotional interactions are destabilized. Here mind and body converge, production and expression of emotion are entangled, and, when applied to emotions, the classic dichotomies – internal/external, private/public, genuine/simulated, true/false – are neither clearly defined, nor constitute perfect oppositions (Damiano, 2009; Damiano et al., 2015). Human–robot interactions, as implemented by SR’s affective loop approach, repudiate the classic thesis that conceives true emotions as internally produced and experienced private events.

## From ‘Dichotomous’ Thinking to ‘Synthetic’ Ethics

### From Condemnation to Impotence

The main weakness of the common view, when used to judge the ethics of SR, is that it leads us to consider all SR’s projects in the same way – as resting on a form of deception and thus as ‘unethical.’ Its only coherent position is a radical condemnation of all social robots, and of all anthropomorphizing technologies, which “should not be allowed into the realm of human relationships.” However, this simple equation between ‘simulation’ and ‘imposture’ is not only unable to account for fundamental ethical differences, but also tends to misrepresent them. Consider, for example, the two following projects: robotic companions built to help autistic children develop social skills, and sex robots that have an integrated ‘rape option.’ In the first case, there are issues concerning the illusion of a reciprocal caring that need to be raised. In the second case, putting the emphasis on ‘fake rape’ may lead to defend rather than to condemn the practice – “What’s wrong with it, it does not hurt anyone?” – but also misses the central difference. In the first case what is aimed at is to empower vulnerable persons, while in the second case the effect is to encourage rape, making it banal and meaningless.

The blanket condemnation of anthropomorphizing technologies and social robots, in turn, condemns ethics to impotence. Social robots will not go away, their development will not stop. What recommendations can a wholesale condemnation provide? What questions can it answer? What dialog is possible between SR and such a form of ethical reflection? Presently, the greatest danger is for SR and ethic reflection on SR to develop in two separate theoretical and epistemological spaces: severing SR from ethical inquiries and reflections that can directly participate to the “new science of human beings” (Parisi, 2014). What SR needs are meta-level ethical analyses leading to guidelines that help it maximize the benefits and minimize the dangers of the construction and integration of artificial social agents in our social ecologies. That is why it is urgent to develop a different form of ethical reflection for SR. An ethics that shares SR’s interactionist embodied approach, and, while recognizing the irreducible (epistemological, phenomenological, operational, etc.) differences that distinguish human–robot from human–human interactions, grants to our exchanges with social robots the status of a new, specific, certainly limited, but genuine, form of social relationships.

### Synthetic Ethics

This form of inquiry will be attentive, and able to respond to aspects of SR’s projects that the dichotomous approaches fail to grasp. Creating anthropomorphizing robots that aid autistic children to develop social skills is miles away from trying to help them by creating the illusion of a reciprocal relationship. Within the different view of emotion sketched above, this project appears as an attempt to address malfunctions in some aspect of these children’s mechanism of social coordination by appealing to other aspects of that same mechanism – in particular to the spontaneous ability at anthropomorphic projections. Such a re-interpretation would constitute the starting point of an ethical inquiry aimed at defining ethical guidelines for this kind of projects, some of which would of course relate to the child–robot relationship, the conditions for it to be genuine and the necessary precautions to be taken.

This new framework provides a significantly different understanding of the second case considered above. From the point of view of affective coordination, sex robots with an integrated ‘rape option’ do not offer to human users to ‘simulate rape.’ Rather they invite users to engage in rape *tout court*, because the proposed practice is embedded in a social context, even though it is one that is mixed – a human–robot social context. As mentioned earlier, human users tend to perceive social robots as interlocutors that break the object/subject divide. They tend to recognize these robots as a new category of inter-actors, with whom they can establish social relationships. If that is the case, raping a robot is still rape, the violation of an agent, even if the artificial agent does not react to this violation the same way a human does. To the extent that robots are truly becoming social agents, participating in our everyday life, developing an embodied interactionist ethics is urgent.

In previous works we introduced, under the name of “synthetic ethics” (Damiano, 2015; Dumouchel and Damiano, 2017), the lineaments of this new approach. Also we argued that the applied anthropomorphism of social robots offers the possibility of deeper self-knowledge, and can be an occasion of moral growth (Coeckelbergh, 2012). The basic idea is to extend the ‘synthetic anthropology’ that is already emergent in SR by applying to ethics in SR the “understanding by building” or synthetic approach (Pfeifer and Scheier, 1999). We refer to “ethics in SR” because the ethical issues concerning social robots do not arise at the border where robots meet society at large. It is not a question of applying new scientific and technological developments. Ethical issues are part and parcel of the very development of this applied anthropomorphism. Synthetic ethics incorporates human–robot interactions in experimental scenarios, analyzing emergent behaviors from an ethical point of view to deepen our knowledge of humans, and of the spontaneous and changing ethics (mores) of human–robot interactions. This knowledge can then be used to review and improve the practices of robots, and to inquire into the ethical (and political) opportunities and dangers of their integration into our social ecologies. The focus should be on the concrete problems that social robots create, or are likely to create, as well as on those issues that can productively be addressed using social robots as research instruments and co-objects of exploration. In short, the applied anthropomorphism of SR can also be a method of inquiry in ethics. This means that the ethics of SR should not be reduced to apply a pre-determined set of rules to an innovative technology. Rather, it has to be conceived as an occasion to enrich our moral knowledge.

Synthetic ethics does not exclude the traditional questions on which focus dichotomous approaches. Rather, it reframes them within a research perspective that views social robots as a means to empower our relationships. How can we build social robots that can work as social connectors, reinforcing human–human relationships, instead of producing isolation and weakening the social bond? How can we design social robots that facilitate, encourage and fortify exchanges among humans, instead of offering the possibility of escaping from the challenges of human–human interaction, and becoming estranged in an effortless world of human–robot interaction? Is it possible to exploit social robots to modify patterns of human behavior, in the direction of ethical growth? Such questions should be, and to some extent already are, part of the applied anthropomorphism of SR. Synthetic ethics is the approach in which these questions need to be raised at the level of the theoretical ideation, design, implementation, and experimental testing of social robots, rather than only addressed from the outside (and after the fact, so to speak) with a pre-established set of ethical rules.

The difference between a ‘dichotomous’ and a ‘synthetic’ approach to the ethics of SR should not be underestimated. The theoretical and epistemological choices we make in order to think, create, understand and regulate social robots will dramatically impact human–robot co-evolution – a mixed social ecology where ethical life may flourish.

## Author Contributions

All authors listed have made a substantial, direct and intellectual contribution to the work, and approved it for publication. The authors elaborated plan and contents of the article together. Each author is responsible for the final form of different parts of the article: Luisa Damiano for Introduction, Section 1 (Social Robotics as Applied Anthropomorphism) and Subsection 2 (Synthetic Ethics) of Section 3 (From ‘Dichotomous Thinking’ to ‘Synthetic Ethics’); Paul Dumouchel for Abstract, Section 2 (Anthropomorphism and Social Coordination) and Subsection 1 (From Condemnation to Impotence) of Section 3 (From ‘Dichotomous Thinking’ to ‘Synthetic Ethics’).

## Conflict of Interest Statement

The authors declare that the research was conducted in the absence of any commercial or financial relationships that could be construed as a potential conflict of interest.

## Footnotes

1 http://www.parorobots.com/

2 https://www.jibo.com/

3 https://spectrum.ieee.org/automaton/robotics/humanoids/meet-affetto

4 http://www.milkeninstitute.org/events/conferences/global-conference/2016/panel-detail/6182

5 https://www.epsrc.ac.uk/research/ourportfolio/themes/engineering/activities/principlesofrobotics/, http://www.europarl.europa.eu/sides/getDoc.do?pubRef=-/ (Principle 4, License for Designers, Point 11).
